# Guiding practitioners through end of life care for people with dementia: The use of heuristics

**DOI:** 10.1371/journal.pone.0206422

**Published:** 2018-11-14

**Authors:** Nathan Davies, Jill Manthorpe, Elizabeth L. Sampson, Kethakie Lamahewa, Jane Wilcock, Rammya Mathew, Steve Iliffe

**Affiliations:** 1 Research Department of Primary Care and Population Health, University College London, London, United Kingdom; 2 Centre for Dementia Palliative Care Research, Marie Curie Palliative Care Research Department, Division of Psychiatry, University College London, London, United Kingdom; 3 Social Care Workforce Research Unit, King’s College London, London, United Kingdom; 4 Barnet Enfield and Haringey Mental Health Trust Liaison Team, North Middlesex University Hospital, London, United Kingdom; Universidade Catolica Portuguesa, PORTUGAL

## Abstract

**Background:**

End of life care (EOLC) for people with dementia can present a multitude of challenges and difficult decisions for practitioners. These challenges may include assessment and management of difficulties with eating and swallowing, responding to agitation, treating pain, and managing recurrent infections. Practitioners sometimes lack both confidence in making end of life decisions and guidance. This study developed an alternative to lengthy guidelines, in the form of heuristics which were tested in clinical settings. The aim of this study was to test the usability and acceptability of a set of heuristics which could be used by practitioners providing EOLC for people with dementia in a variety of clinical and care settings.

**Methods:**

A three phase co-design process was adopted: 1) Synthesis of evidence and outputs from interviews and focus groups with family carers and practitioners, by a co-design group, to develop heuristics; 2) Testing of the heuristics in five clinical or care settings for six months; 3) Evaluation of the heuristics at three and six months using qualitative individual and group interviews.

**Results:**

Four heuristics were developed covering: eating and swallowing difficulties, agitation and restlessness, reviewing treatment and interventions at the end of life, and providing routine care. The five sites reported that the heuristics were simple and easy to use, comprehensive, and made implicit, tacit knowledge explicit. Four themes emerged from the qualitative evaluation: authority and permission; synthesis of best practice; providing a structure and breaking down complexity; and reassurance and instilling confidence.

**Conclusion:**

Use of heuristics is a novel approach to end of life decision making in dementia which can be useful to both experienced and junior members of staff making decisions. Heuristics are a practical tool which could overcome a lack of care pathways and direct guidance in end of life care for people with dementia.

## Introduction

Dementia is now the most common cause of death in England and Wales [1]. There is no known disease modifying treatment for the syndrome. End of life care (EOLC) for people with dementia is becoming one of the major priorities for health and care policy internationally, as reflected in many national dementia strategies in several countries across the world [2, 3].

EOLC for people with dementia is likely to be complex for a number of reasons; cognitive impairment is progressive but many people with dementia are also frail and have multiple comorbidities [4]. Towards the end of life there may be many complications which can be distressing and create dilemmas for practitioners and family members about what to do [4]. Problems such as difficulties with swallowing and therefore difficulties with eating, drinking and taking oral medication; agitation; and a diminished immune response leading to greater susceptibility to infections, all create different and difficult/complex options which need to be decided between. These can include Do Not Attempt Resuscitation (DNAR) orders, the use of feeding tubes and whether to provide intravenous antibiotics [5]. The most frequent causes of death for someone with dementia include cachexia and dehydration, respiratory infections and cardiovascular disorders [6, 7].

There is currently a lack of guidance for practitioners working with people with dementia at the end of life. Dementia guidelines often have little discussion of end of life problems and palliative care guidelines are often aimed at people with cancer [8–10]. A white paper from the European Association for Palliative Care (EAPC) was published in 2014, it offers more high-level guidance about palliative care for people with dementia, covering 11 core domains [11]. Despite this there is little to guide day-to-day decision making for practitioners caring for someone with dementia at the end of life. Many practitioners caring for someone with dementia may not have much experience of either caring for someone with dementia, or caring for someone who is dying. When faced with a combination of the two they may lack confidence. Previously one of the main documents practitioners referred to at the end of life to guide care was the Liverpool Care Pathway, mainly for the final 48 hours of life. However the media attention, criticism and subsequent removal of the Liverpool Care Pathway in the UK has left practitioners feeling vulnerable and potentially even less confident in EOLC [12, 13].

Clinical decision-can be a complex process as it often takes place acutely, has to take into account many uncertainties, and when knowledge, time, and resources are limited, which adds to the complexity of providing end of life care for people with dementia[14]. Guidelines which attempt to support clinical decision making are often long documents which are not easily accessible, are not sufficiently evidence-based or are based on low quality evidence and become obsolete quickly [15, 16]. Furthermore, practitioners are less influenced by guidelines than they are by rules-of-thumb (heuristics) [17]. Heuristics are broad principles, which can be applied in complex situations, prompt thinking, and lead to action [17, 18]. They are simple decision strategies that ignore part of the available information, basing decisions on only a few relevant predictors. Heuristics can be useful in health care settings because of their surprising accuracy, transparency, and wide accessibility, as well as their low costs and speed of use [19].

An example heuristic (expressed as an acronym) is used to identify those suspected of having a stroke; FAST (**F**acial drooping, **A**rm Weakness, **S**peech Difficulties, **T**ime to call emergency services) [20]. A recent review found there was a lack of heuristics in dementia care [18].

The aim of this study was to test the usability and acceptability of a set of heuristics which could be used by practitioners providing EOLC for people with dementia in a variety of clinical and care settings.

## Methods

### Ethical considerations

Ethical approval for the study was obtained from both University College London (ID:3344/003) and from the National Research Ethics Service London—Camden and King’s Cross (ID:15/LO/0156), and local approvals were granted where needed. All participants provided informed written consent prior to participating in group or individual interviews.

### Design

This study adopted a three-phase co-design process:

Phase1) Outputs from qualitative interviews and focus groups with family carers and practitioners, and findings from a literature review were synthesised by a co-design group to develop a toolkit of heuristics;
Phase 2) Field testing of the heuristic toolkit for 6 months in five different settings;
Phase 3) Evaluation of the heuristic toolkit, with individual and group interviews.

**Fig 1:**
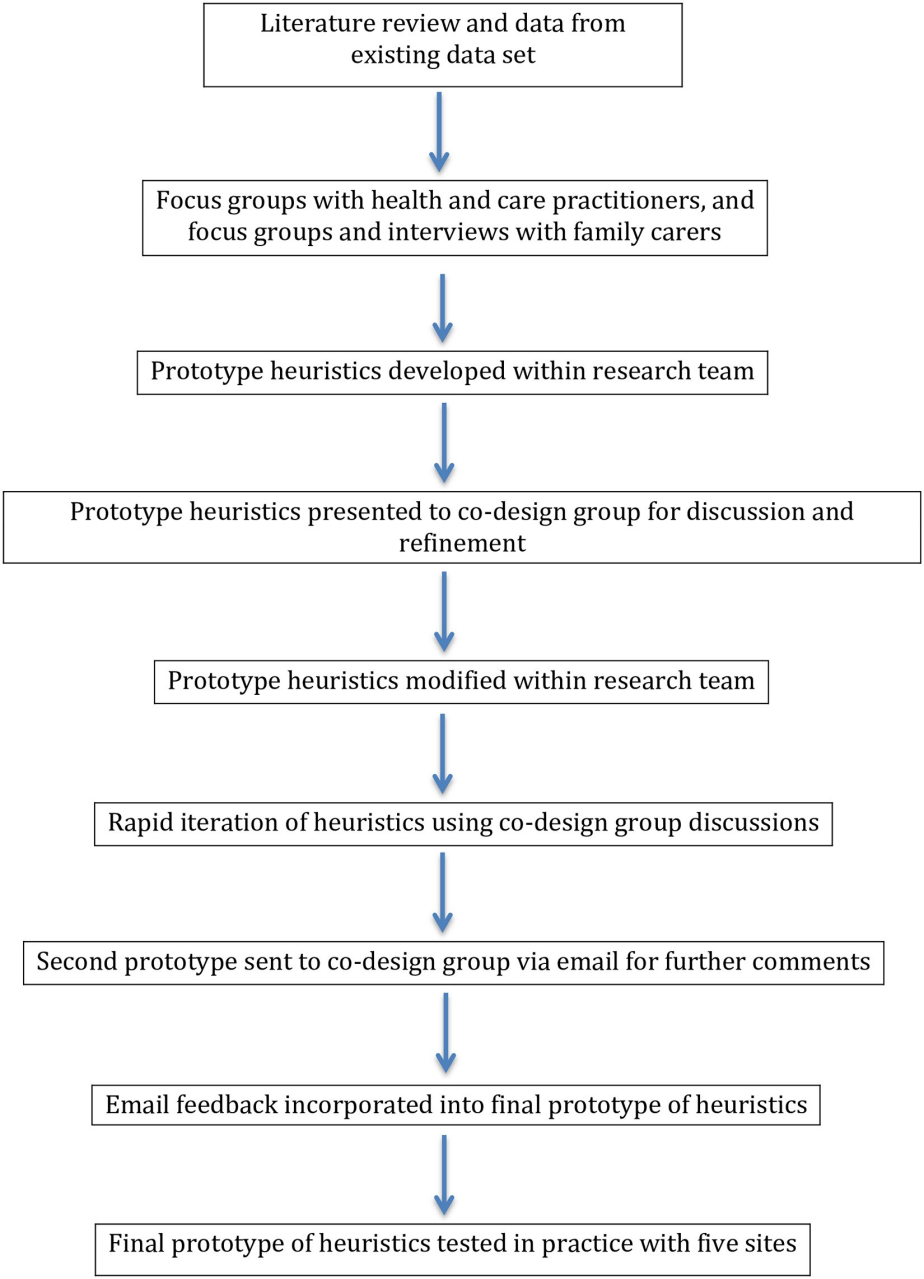
Overview of procedure (previously published as part of methodology paper for this study [28].

Co-design is a technique developed from the technology design and product development industry [21] and has benefits for developing or redesigning health and care services [22–25]. It has been adopted by health and care based research and systems as ‘experienced based co-design’. It is an approach which involves gathering experiences from patients and staff through in-depth interviewing, observations and group discussions [26], (as in phase 1 of this study). In phase 2 we continued with this experience based co-design approach to develop the heuristics. We constructed a co-design group tasked interpreting the findings from phase 1 and developing a toolkit of heuristics. The group consisted of health and social care practitioners (palliative care consultant, two GPs, Admiral nurse (specialist dementia nurse), social care professional, two geriatricians, and a community nurse), four family carers and members of the research team with backgrounds in psychology, social care, old age psychiatry, anthropology, and general practice. An overview can be seen in Fig 1.

The full methods for this study and the phase 1 development of the heuristics are published [27, 28]; this article presents the findings from phase 2 and phase 3.

### Settings/Participants

In phase 2 five clinical and care settings were recruited consisting of urban and semi-rural services across London and Essex: one complex care acute hospital ward with a large proportion of patients with dementia, one general practice, one community nursing team, and two palliative care community teams.

The hospital ward was based in a district general hospital. The hospital is based in an urban part of Essex, England, which is in the second most deprived quintile nationally, and had previously had minimal engagement in research. The two palliative care community teams were based in North and South London which serve areas of varied social deprivation. Both teams had an increasing caseload of people with dementia, and one of these teams had a dementia nurse specialist. Both palliative care teams are research active. The community nursing team was a social enterprise providing nursing care to a semi-rural population in Essex with low levels of social deprivation. The nursing team had previously not engaged in research. The general practice was a research active practice in North London, serving several care homes, encompassing a patient list from a mixture of low and high socially deprived areas.

In Phase 3 practitioners were recruited in each of the five sites who had used the heuristics in providing care for people with dementia at the end of life.

### Recruitment

Sites were identified through the Dementia and Neurodegenerative Diseases Research Network (DeNDRoN) co-ordinating centre, the comprehensive Local Research Network (CLRN), and known contacts of the research team (adopting a snowballing technique) [29].

### Procedure

The findings of the qualitative interviews and focus groups with family carers and practitioners were presented to the co-design group described above. The co-design group were tasked with interpreting the evidence and developing a toolkit of heuristics. Further details of the development of the heuristic toolkit are published elsewhere [28]; an overview can be seen in Fig 1.

Following the development process, potential sites were identified and invited to join the study by the research team, CLRN or DeNDRoN in writing via email. Interested sites were followed up by a member of the research team (initials to be inserted after review) and provided with more detail about the study and the heuristics to be tested. An initial introductory meeting was arranged at each site where the heuristics were presented to the teams as a group. Practitioners were asked to use the heuristics as a framework for providing care for up to 10 people with dementia for a period of 6 months.

A lead member of the team was identified in each of the sites to provide a point of contact for the research team and act as a champion for the use of the heuristics. Staff using the heuristics in each site participated in a group interview after 3 months of using the heuristic toolkit. These interviews gave them an opportunity to suggest any changes to the heuristics. Following a small number of changes made by the research team in conjunction with the co-design group (28), the modified heuristics were introduced to the sites once again through a meeting with all staff or the champion in that site, highlighting the changes in the heuristics. Staff in the sites were asked to continue using the heuristics for a further 3 months.

At 6 months staff in all sites were interviewed individually to allow for a more in-depth understanding of how participants used and experienced the heuristics in practice. Interviews were audio recorded and transcribed verbatim. Interviews focussed on the use of the heuristics in the site, exploring how they were most useful, acceptability among the team, suggestions for further iterations, and finally the advantages and disadvantages of using the heuristics.

### Analysis

To inform further iterations of the heuristics the interviews at both follow-up time points (3 and 6 months) were summarised by three researchers (ND, KL, RM) from the research team using a rapid thematic analysis approach [30]. They presented these summaries to other members of the research team (SI, JW, JM) to further discuss the findings and reach a consensus on summaries. The summaries were used to conduct further iterations to the heuristics and provide a finalised toolkit.

A thorough and final thematic analysis was conducted on the interviews at six months to understand the usability and acceptability of the heuristics. A team approach to analysis was adopting increasing rigour in the analysis [31]. Two researchers (ND, KL) independently coded two transcripts and met to discuss a coding strategy for all the interviews. The remainder of the interviews were coded by (ND) and a random selection were reviewed by (SI). Following coding two researchers (ND, SI) met to discuss emerging themes, and revised these iteratively. Themes were presented and discussed among all members of the research team, relationships among the themes were explored and discussed, searching for negative or deviant cases, increasing the rigour [31]. The analysis team, consisting of a multidisciplinary research including psychologists, health service researchers, social care expert, and general practitioners, added to the depth of discussion and interpretation of findings.

### Findings

Four heuristics were developed covering the main topics considered challenging at the end of life: eating and swallowing difficulties, agitation/restlessness, reviewing treatment and interventions at the end of life, and providing routine care at the end of life. The heuristic toolkit is aimed at maximising comfort, reducing distress and maintaining dignity. These topics were finalised following interviews with practitioners and family carers in phase one and a synthesis of evidence [18, 28]. The development process and the prototypes of the initial heuristics have been presented in a previous article [28]. The current article presents an evaluation of the heuristics from the perspective of professionals who used them in practice and the finalised heuristics following evaluation and further discussions with the co-design group.

### Finalised heuristics

The section presents the finalised heuristics following 6 months testing in practice an evaluation with the professionals who used them in practice. The main changes to the heuristics were focussed on wording and reducing complexity of the flowcharts. With some heuristics further emphasis was placed on particular aspects of care, for example, ensuring in agitation and restlessness that medication was not the first option under no identifiable cause. Only one heuristic changed its name ‘Reviewing treatment and interventions at the end of life’ was originally ‘ending life sustaining treatment’. The evaluation considered this to be too negative and furthermore ambiguous as to which treatments would be included.

#### Eating and swallowing difficulties

**Fig 2:**
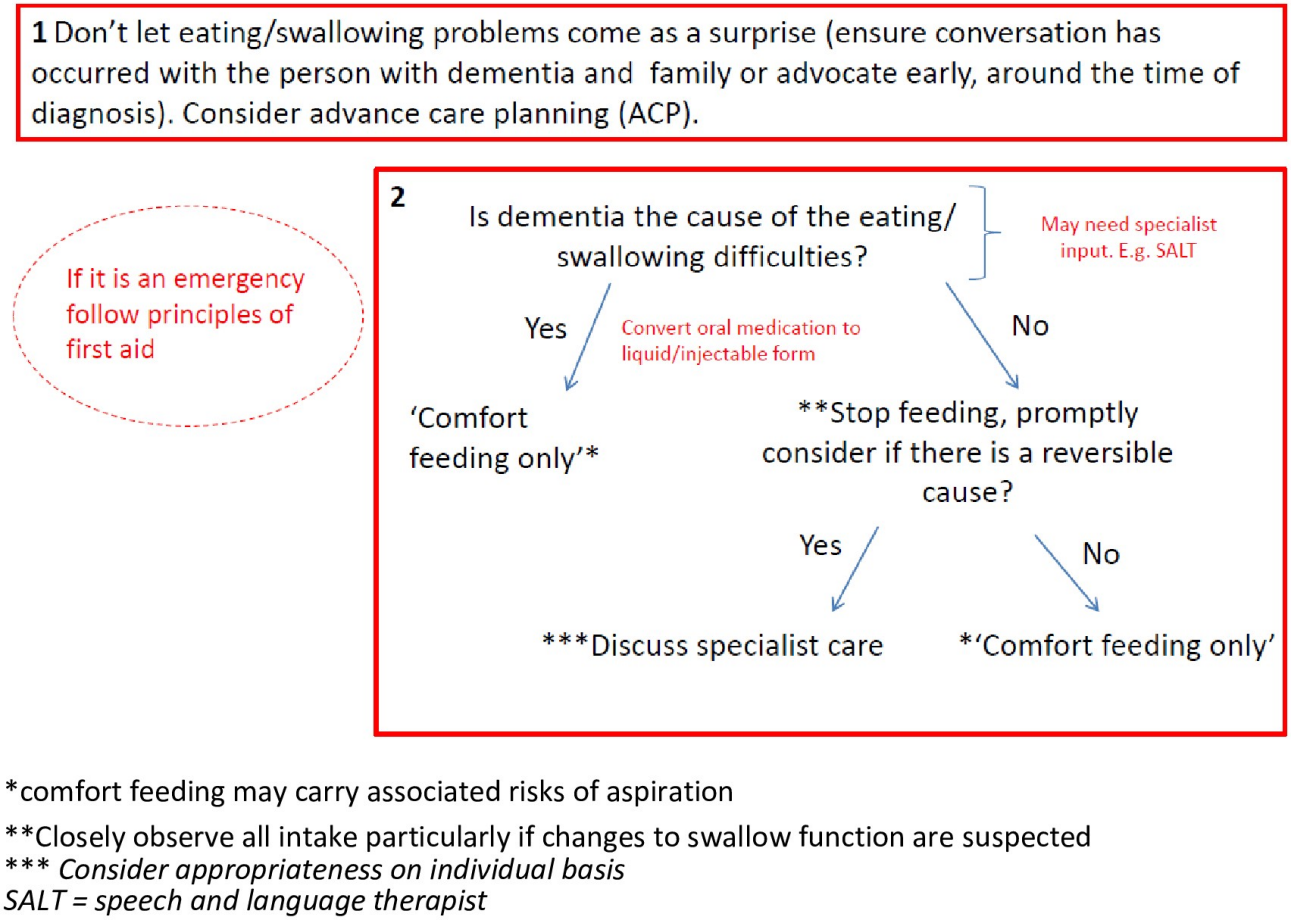
Eating and swallowing difficulties. *comfort feeding may carry associated risks of aspiration **Closely observe all intake particularly if changes to swallow function are suspected *** Consider appropriateness on individual basis SALT = speech and language therapist. Reprinted from Davies and Iliffe under a CC BY license, with permission from Davies and Iliffe, original copyright 2016.

The heuristic on eating and swallowing difficulties had two rules (Fig 2). The first takes a proactive approach ensuring eating and swallowing difficulties do not come as a surprise, encouraging advance care planning and early discussions. The second rule focusses on when the difficulties occur and introduces the idea of ‘comfort-feeding’ as well as treating any potential reversible causes. The text in the balloon in the top left corner is a caveat about emergency situations—in this case choking.

#### Agitation and restlessness

**Fig 3:**
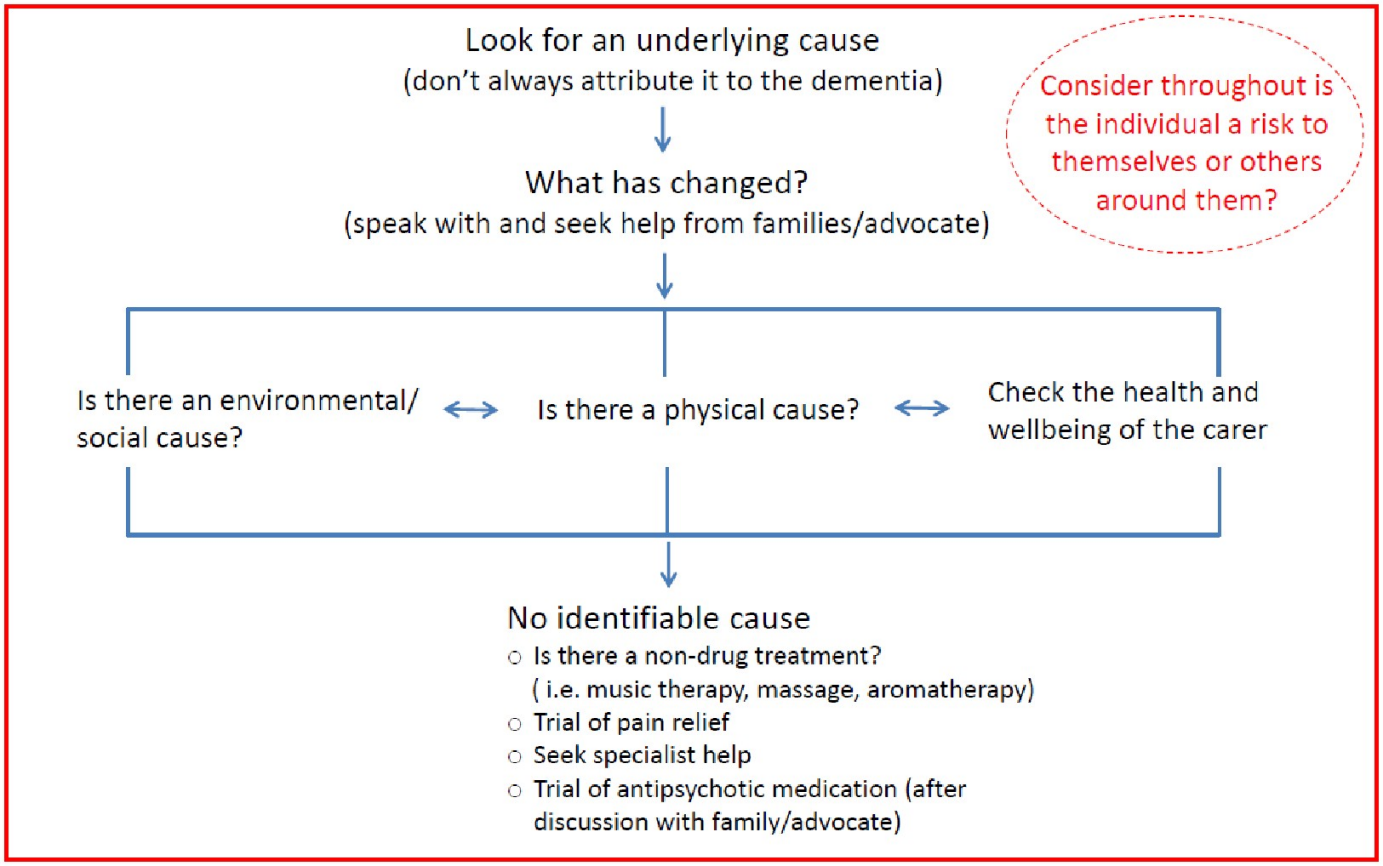
Agitation or restlessness. Reprinted from Davies and Iliffe under a CC BY license, with permission from Davies and Iliffe, original copyright 2016.

The agitation/restlessness heuristic encourages a holistic approach (Fig 3). Firstly, the key message is to ensure that the agitation or restlessness is not simply attributed to the dementia. There are three main areas for consideration; the environment, physical causes, and carers’ health and wellbeing. All three are to be considered in parallel and no one of them is more important than the other. Bi-directional arrows show how possible causes of restlessness should not be considered in isolation but as a spectrum to be reviewed and reconsidered when assessing a person with dementia who is agitated or restless.

#### Reviewing treatment and interventions at the end of life

**Fig 4:**
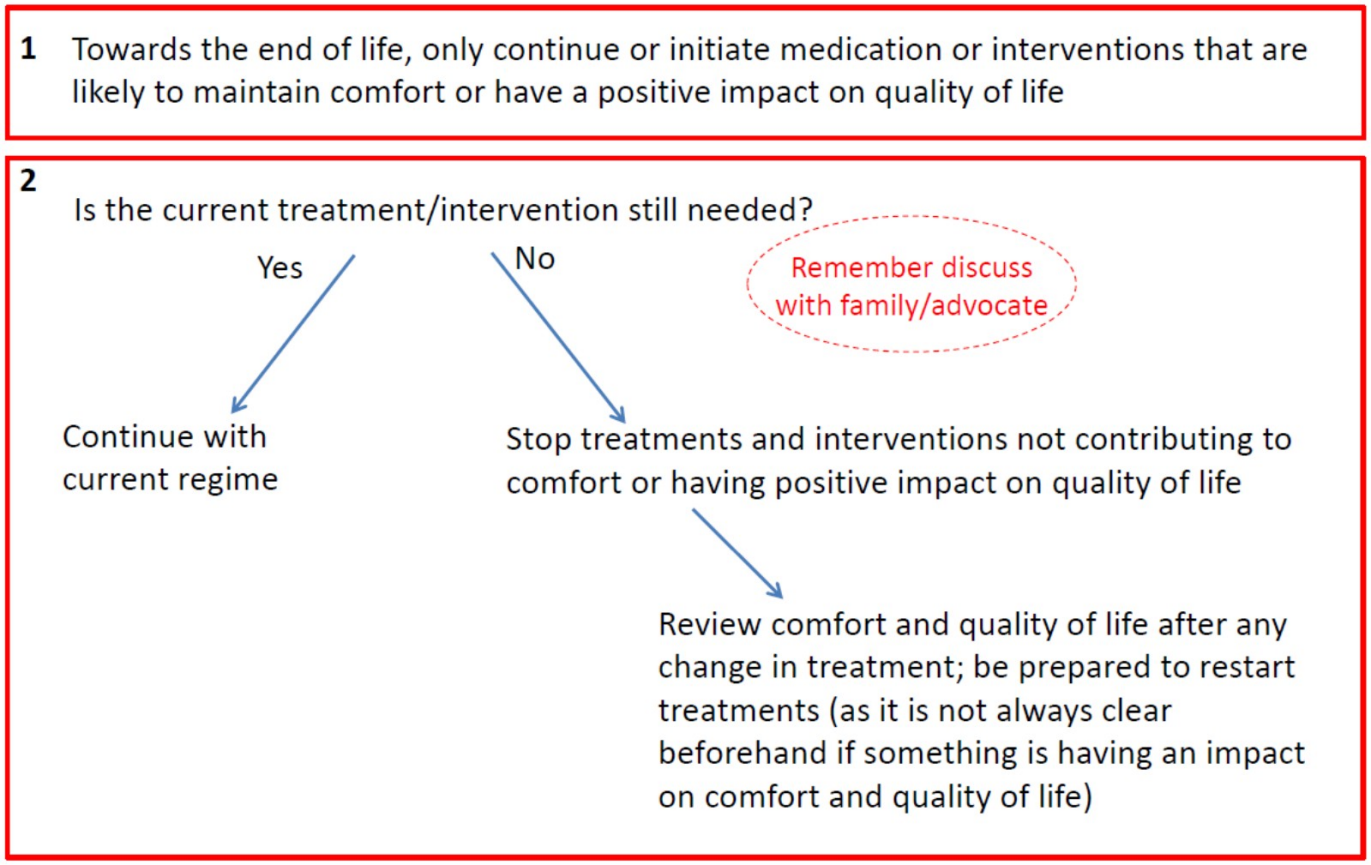
Reviewing treatment and interventions at the end of life. Reprinted from Davies and Iliffe under a CC BY license, with permission from Davies and Iliffe, original copyright 2016.

The third heuristic covers reviewing treatment and interventions at the end of life and prompts practitioners to consider their benefits for quality of life and comfort (Fig 4). It asks the simple question: *is the current treatment still needed*? If a treatment is not needed then the heuristic suggests stopping any interventions that are not having a positive impact on quality of life, but also to regularly review any changes and be prepared to restart treatments if appropriate.

#### Providing routine care at the end of life

**Fig 5:**
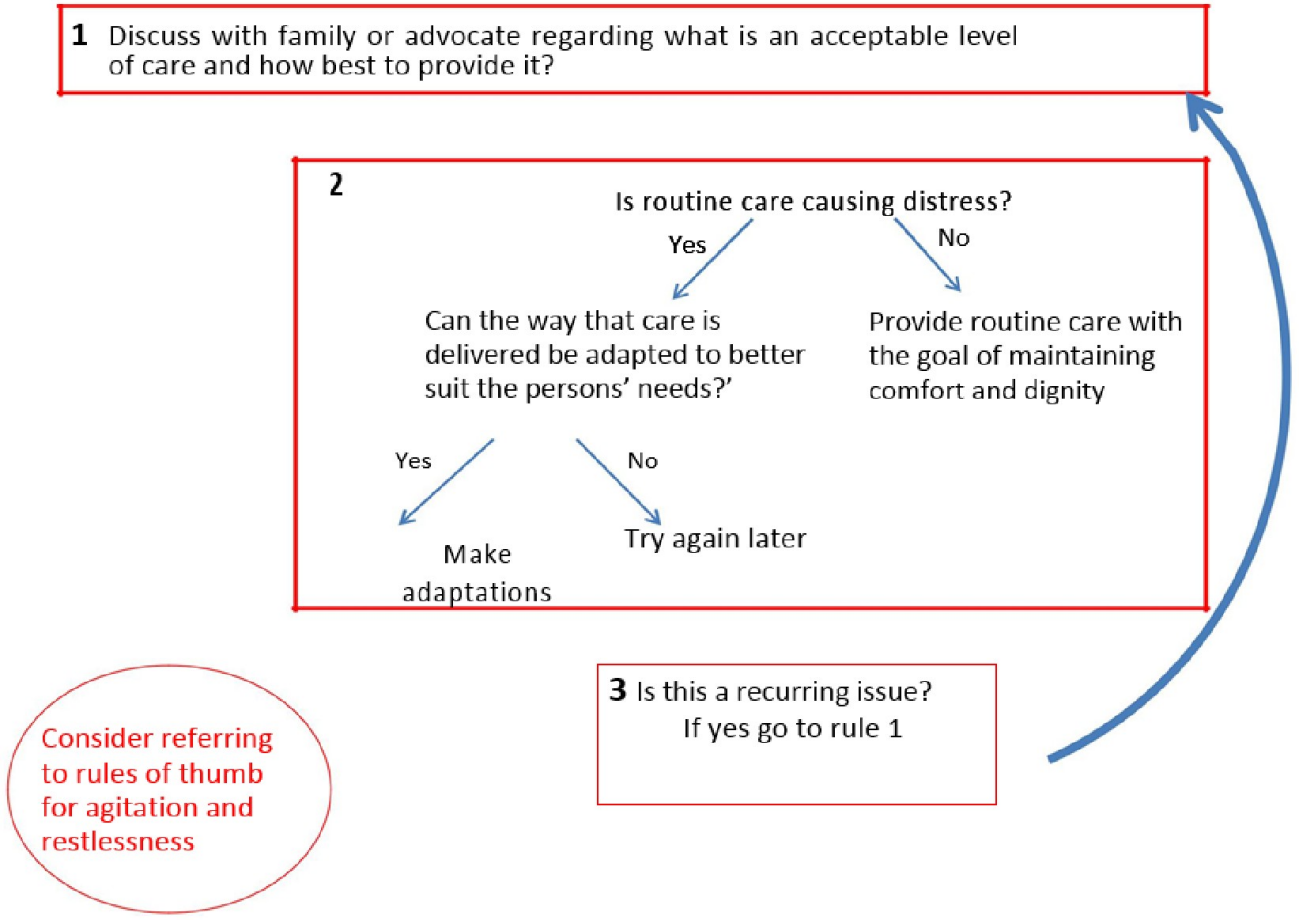
Providing routine care at the end of life. Reprinted from Davies and Iliffe under a CC BY license, with permission from Davies and Iliffe, original copyright 2016.

Finally, providing routine care at the end of life focusses on the final days to hours of life (Fig 5). Routine care in this heuristic includes care which is aimed at improving comfort but may not be essential, for example changing bed sheets which are still clean or bathing a person because it is part of routine activity. The heuristic prompts practitioners to ensure such care interventions have positive impacts on quality of life and to discuss an acceptable level of care with families/advocates.

### Evaluation of heuristics in practice

A total of 19 members of staff were interviewed for up to one hour each, from the five participating sites, covering a range of roles including; dementia nurse specialists, community matrons (experienced senior nurses who work with patients with serious long term or complex conditions in a community setting, including providing direct care in addition to planning and organising care), GPs, palliative care nurses, ward nurses and a ward manager (see Table 1). Four themes emerged from the interviews: Authority and permission; Synthesis of best practice; Providing a structure and breaking down complexity; and Reassurance and instilling confidence. Each theme is described below and in Table 2.

**Table 1:** Participant characteristics.

Participant ID	Type of site	Gender	Role	Experience of working with people with dementia at the end of life
DS350044	Community nursing	Female	Nurse Practitioner/ Community Matron	25 years
DS350045	Hospital	Female	Hospital nurse	Junior
DS350046	Hospital	Female	Nurse Ward Manager	20 years of experience-
DS350047	Hospital	Male	staff nurse	5 years of experience
DS350048	Hospital	Female	3^rd^ Year Student nurse	-
DS350049	Hospital	Male	Hospital matron	30 years
DS350050	Community palliative care	-	Clinical nurse specialist in palliative care	11/12 years of experience
DS350051	Community palliative care	Female	Lead nurse Palliative Care Team	Exact experience in years not provided—senior role
DS350052	Community palliative care	Female	Palliative care nurse	Around 5 years of experience
DS350053	Community palliative care	Female	Dementia nurse specialist,	-
DS350054	Community palliative care	Female	Dementia nurse specialist	Dementia specialist nurse for 1 year (unsure about prior experience)
DS350055	Community palliative care	Female	Ward sister	Ward sister for 2 years (unsure about prior experience)
DS350056	Community nursing	Female	Community Matron	This role for year and a half. Prior work as specialist heart failure nurse for six years.
DS350057	Community nursing	Female	Facilitator for end of life care (education)	District nurse background (senior)
DS350058	General practice	Male	GP partner	Not known
DS350059	Community nursing	Female		30 years’ experience. Last 8 years end of life work, so more specialist.
DS350060	Community nursing	Female	Community Matron	Senior nurse, years of experience not known
DS350061	Community nursing	Female	Community Matron	Senior nurse, years of experience not known
DS350062	General practice	Female	GP partner	

**Table 2:** Summary of themes.

Themes	Summary
Authority and permission Feeling of permission to challenge the norm; Feeling of possessing some authority in discussion with families; Challenging colleagues, Empowering families; Empowering staff	The heuristics provided practitioners with a source of authority to guide discussions with colleagues and families. They were also seen as a potential source of empowerment if used by family carers in the future.
Synthesis of best practice	For many practitioners the heuristics provided a simple representation of what they already did as part of their practice. They offered a clear representation of the tacit knowledge which had been developed over many years of experience.
Providing a structure and breaking down complexity	Practitioners felt the heuristics offered a clear and simple approach to decision making and approaching care for people with dementia at the end of life, breaking down the complexity.
Reassurance and instilling confidence	Practitioners observed that this was often a challenging and complex field and the heuristics provided a source of reassurance. The toolkit could be used for both reassuring experienced practitioners but also upskilling those with a lack of experience in providing care for people with dementia at the end of life.

#### Authority and permission

Practitioners reported feeling that the heuristics offered them a source of authority or confidence with their own expertise when caring for someone with dementia. This authority could operate in a variety of ways including: authority to challenge the norm; authority in discussion with families; challenging colleagues, empowering families; and empowering staff.

Feeling of permission to challenge the norm: Some participants acknowledged that EOLC was often delivered in a routine manner because this was the ‘norm’ and how things had always been done. The heuristic gave permission to challenge the care practices and decisions:

*“This first section (section 1 of* Fig 3*) I think is absolutely right*. *What (Why) are you still doing blood pressure*? *What are you still taking blood for*? *What are you hoping to achieve*? *If somebody is actually actively dying and they really are*, *what do you think you are going to do*?*”*

(DS350056, Community Matron)

*“one of the things that nurses and families get really*, *really obsessed (about) is the patients being clean*—*whilst I totally agree that every patient should have their dignity of being clean*, *if that’s what they want*, *is it actually all right to leave them [*…*] I have never seen anything written before in that if they say*, *no*, *leave them*. *I think that’s really helpful*, *really helpful*.*”*

(DS350059, End of Life Care Facilitator)

Feeling of possessing some authority in discussion with families: Having heuristics written out as a resource provided a source of authority which practitioners could show to families when discussing their thoughts on treatment and/or care:

*“They (family) did begin to understand because it*’*s all set out*, *I’m not suggesting it off the top of my head but it*’*s set out on paper and it*’*s backed (up)”*

*(DS350063*, *Community Matron)*

Challenging colleagues: The experience of using the heuristics highlighted an important role for the nurse participants; the need to question doctors to ask if a certain treatment or care action was really beneficial to the patient:

*“It’s quite good sometimes because like I say*, *sometimes you have junior nursing staff that need to challenge junior doctors or senior doctors*. *Sometimes*, *it’s quite useful to actually say*, *well actually*, *there you go*, *read it*…*”*

(DS350049, Senior Hospital Nurse)

Empowering families: In offering the ability to challenge and breach the norms in order to achieve best care for the patient and their family, practitioners felt that what they were describing in the heuristics was a potential source of empowerment, in particular empowerment for families:

*“This is a really powerful tool I think to have*, *because I think often*, *because this is our thing*, *you know*, *[*…*] we speak to the GPs and say that we think it’s a UTI (urinary tract infection) and they will probably listen to us*. *But I think for lots of other people that’s not always the case*. *[*…*] for carers being able to say*, *I’ve looked at this*, *this*, *this*, *this and it’s none of these things would maybe be really helpful*. *I would really*, *really like that*.*”*

(DS350054, Dementia Nurse Specialist)

Empowering staff: The heuristics could also be a source of empowerment for practitioners, providing them with the confidence to use the tool and trust their own knowledge and expertise:

*“I like the fact in here that it’s saying*, *only refer to speech and language therapy if it’s not just thought to be associated with the dementia and actually thought to be something else there*. *I think that that’s kind of quite empowering for people as well to use their judgement and say*, *right*, *we don’t need a referral*.*”*

(DS350054, Dementia Nurse Specialist)

#### Synthesis of best practice

The teams that were trialling the heuristics were experienced in providing EOLC for people with dementia, which enabled the research team to be confident the content was accurate, thorough and comprehensive. Many felt that the heuristics were simply a representation of what they already did, and so were a synthesis of best practice:

*“[*…*] actually it’s (heuristics) just what we do and what we want to do*.*”*

(DS350049, Senior Nurse)

The heuristics were seen as a fusion of experience and knowledge which had been developed over many years, displayed in a simple, easy to understand format. For many they were nothing new, however they could act as a refresher, making implicit knowledge and experience explicit for everyone to see and share:

*“It’s a very good tool*. *I think even for palliative care who have been doing it*, *but without actually having guidelines to guide us through it*. *It’s something that is almost ingrained in us*. *But actually*, *seeing it there visually*, *it has been very helpful*. *Yes*, *as a teaching tool*, *excellent*.*”*

(DS350050, Palliative Care Clinical Nurse Specialist)

The heuristics enabled practitioners to reflect on their decisions before acting on them, but also for them to reflect on their actions and to identify aspects of care which could be improved on in the future:

*“But still this is helpful in just initially going through it*, *but then looking back after a situation and thinking*, *could we have done anything differently*?*”*

(DS350053, Dementia Nurse Specialist)

Those with more experience also felt it was also a useful resource which they could use with junior members of their team or those who had little experience in dementia and/or EOLC:

*“So the people who aren’t used to working with end of life patients*, *I would use this as a bit of recap training for every couple of months*.*”*

(DS350047, Nurse)

However, some participants suggested that the heuristics may need to be setting specific, to account for different practices, for example, between community and hospital care:

*“the NG (nasogastric) tube is not (for community)*, *[*…*] is not what I would consider really*, *although I generally would*, *as you say on here*, *discuss specialist care [*…*]*.*this is more for hospitals isn’t it*, *really*?*”*

(DS350058, GP)

#### Providing a structure and breaking down complexity

Participants described the heuristics as offering a structure to their role, thought processes, discussions with families and ultimately decisions:

*“When I am having the discussion with them (families) about end of life and the planning for the future and got more of a structure to what I am saying*, *which I am finding helpful”*

(DS350052, Palliative Care Nurse)

Practitioners appreciated the simplicity of the heuristics both in concept and in design, and their logical flow. They considered that the tool provided a simple representation of the common complex decisions which can arise at the end of life for someone with dementia. In breaking down the complexity practitioners discussed how they were able to broaden their thoughts and think about other non-physical aspects of care, which they may not have considered previously:

*“I think the layout is good*. *It’s nice and clear*. *They are very simple aren’t they but I think that’s the simpler the better really isn’t it*. *They are more likely to use it rather than go on*.”

(DS350055, Sister)

*“We sort of looked at it outside the box*.*”*

(DS350057, Community Matron)

Despite providing structure practitioners felt the heuristics still enabled them to deliver person centred care:

“It was still about keeping the patient central”

(DS350056, Community Matron)

#### Reassurance and instilling confidence

For many, including those who were very experienced, providing dementia care remains difficult and full of uncertainty, and providing EOLC is similarly difficult regardless of underlying condition or disease. Moreover, the practitioners in this study were providing EOLC for people with dementia, two complex and difficult areas of care. Practitioners often talked about this situation as ‘scary’ and needing reassurance that they were doing the right thing. The toolkit offered a source of reassurance when they were unsure and wanted to check:

*“I think it just gives probably the member of staff confidence to discuss issues*, *especially if DNAR (do not attempt resuscitation orders) is in there because it will give them the confidence to discuss it*. *We do it for a lot of our palliative patients*, *automatically*. *With dementia*, *not so much*. *[*…*] It gives staff confidence to discuss it*, *because it’s there in black and white*.*”*

(DS350061, Community matron)

*“I think it’s very simple but it’s reassuring and in this day and age when people are frightened of being sued or criticised*, *it’s really important to have reassurance that you’re doing the right thing [*…*]”*

(DS350063, Community matron)

Some community practitioners thought this tool would be particularly helpful for those working more infrequently with end of life patients in the community:

*“It won’t be really used by the advanced practitioners probably like palliative care*. *But I think it certainly would be useful for people with*, *people who maybe from primary care*, *doctors*, *if they are not so used to looking after patients and haven’t had a lot of experience with patients with dementia and primary care*. *Maybe looking after a care home or sheltered housing or something where people are more likely to be end of life care*.*”*

(DS350058, GP)

Others saw it as a potentially temporary way of ‘upskilling’ staff in situations which required immediate attention when no experienced practitioner was available:

*“I can’t think of any care home that wouldn’t want a resource like this*. *That they can just pull out and have a look at*. *Obviously they’ll be referring on to matrons or district nurses anyway but*, *initially*, *when it does first happens and to look back and reassure themselves”*

(DS350063, Community matron)

## Discussion

This study has shown that, using a co-design approach, it is possible to frame common and difficult decisions about dementia care in simple terms. We have developed a toolkit of four heuristics covering; eating and swallowing difficulties, agitation and restlessness, reviewing treatment and interventions at the end of life, and providing routine care. The process of developing these heuristics made the knowledge which is often implicit, and tacit to those experienced, explicit for everyone to understand. Most practitioners do not think consciously about heuristics. Becoming more aware of them and developing a common vocabulary may help them to be used effectively [32].

It is an empirical rather than an *a-priori* assumption about how well cognitive heuristics can function in an uncertain environment [33]. This study opens up the exploration of heuristic use in the care of people with dementia at the end of life. The toolkit of heuristics developed in this project has been incorporated into the training programme of the UK Alzheimer’s Society for its community workers and within a general hospital within England, suggesting that the evaluation we carried out was sufficiently convincing to prompt wider use of the heuristics.

A recent review has highlighted the lack of guidance and support for practitioners delivering EOLC for people with dementia [11]. Dementia, like many other long term conditions, often brings with it a large element of complexity and uncertainty around symptoms and the dying trajectory [34] The heuristics offer a source not only of structure and guidance for practitioners delivering care, but also reassurance and confidence which many are lacking [12, 13].

Having greater confidence underpinned by the heuristics potentially empowers not only practitioners but also family carers, allowing them to understand and challenge the decisions of others about care. The need to challenge others’ decisions has been highlighted in the literature, in particular the need for family carers to challenge professionals [35, 36]. However, this study suggests that practitioners also need to be prepared to challenge one another when acting in the best interests of the person with dementia, and the heuristics offer a vehicle to do this. For example, nurses challenging doctors in hospital settings. Many practitioners may find challenging uncomfortable but professional codes of practice may declare it to be their responsibility.

### Implications for clinical practice and training

Heuristics of the kind described in this article may have different use or value to varied groups of practitioners. The heuristics have been described in the current study for the use in the induction of new staff, and in the speedier transformation of novices into competent practitioners.

The use of heuristics in EOLC for people with dementia has highlighted that many care decisions are reactive, partly because many people with dementia lack a care plan to which they have had input [37]. Although the heuristics in the current study were initially developed to help practitioners with reactive responses, the findings suggest that the heuristics may also be a useful tool for care planning. They could be used by practitioners to guide on-going care planning discussions with families and people with dementia. Many people with dementia often find it difficult emotionally to engage in discussions in the earlier stages of dementia around their future and care. The heuristics offer a tool to engage in conversations with both the individual and to those caring for them at any point in the dementia trajectory.

The removal of the Liverpool Care Pathway created a new problem of what can be used to guide practitioners to care for people at the end of life, with reviews ruling out the development and use of future pathways [38]. The heuristics in this study help overcome this problem by offering an alternative to a care pathway, and provide a more practical approach to guidance. The heuristics in this study are specific to end of life care for people with dementia, also addressing a concern that the Liverpool Care Pathway was not well suited to caring for people with dementia.

### Strengths and limitations

To the best of our knowledge this study is the first to develop guidance for responding to the clinical and care situations experienced by people with dementia at the end of life, in the form of heuristics. This article builds on the previous published articles of this study [27, 28], providing the finalised heuristics and an evaluation of them in practice. The positive response to them from those who used them in practice suggests that the heuristics capture useful thinking in a format useable in fast moving situations. However, there is a risk of bias in that those who take part in this kind of research and development studies are not necessarily representative of a wider professional group or carer population.

Heuristics can be associated with risks. For example, when using heuristics practitioners may jump to a conclusion that might not be accurate. This may be based on an initial impression, or be over-influenced by salient or recent events that happen to be more available and accessible to their working memory and intuition [32]. This is why heuristics need to be designed and tested in real-world settings and within multi-disciplinary work environments, and to be published for wider discussion.

It may be that this study’s focus on decision-making does not adequately address the known barriers and challenges to the delivery of high quality EOLC for people with dementia [39–42]. Other perspectives may emphasise organisational barriers such as the lack of connection between services, the risks of services becoming mechanistic, personal challenges about what constitutes sufficient training, and skill in the negotiation of risk and fear that those working with people with dementia need. We do not discount these perspectives, but see heuristics as a way of engaging with them, and interpret the uptake of the heuristic toolkit as evidence that it meets a need. Finally, the heuristics may not be appropriate for all cases for example in cases were the families may wish to continue to pursue life prolonging treatment, however the heuristics may be useful as a means of discussing with families appropriate goals of care.

### Future research

We hope to expand our understanding of heuristics, and further modify our toolkit, by testing their utility in wider settings, especially in care homes. Family carers may also wish for greater support in making decisions, in particular at home or when their relative is in a care home. This study has helped to identify what decisions need to be made towards the end of life for someone with dementia; however it has focussed on the decisions that practitioners make rather than family carers. In conducting this work, carers consulted reported that the decisions they make are sometimes different and more work is needed to explore these.

The methodologies needed to further develop and evaluate heuristics appear complex and may need to be developed for real time practice through observation or role play. Evaluation of EOLC practice needs to be set in its broader political, cultural and organisational contexts which are difficult to control. Case studies, participatory action research, and before-and-after studies are useful ways of assessing the impact of contextual factors on EOLC, where experimental studies like Randomised Controlled Trials may not be feasible [43].

## Conclusions

This study has developed a finalised practical toolkit of heuristics which can be used across settings by practitioners providing end of life care for people with dementia. The heuristics offer a novel approach to decision making for practitioners, which appear acceptable to practitioners who provide EOLC for people with dementia. The co-design methodology ensured the heuristics are grounded in both the experiences of family carers and practitioners. Heuristics are not without their limitations; however, this study suggests they provide a source of confidence and reassurance for a time period and circumstance which practitioners find challenging. Heuristics provide a practical toolkit which can be utilised alongside existing guidelines.
